# Addiction, the Concept of Disorder, and Pathways to Harm: Comment on Levy

**DOI:** 10.3389/fpsyt.2013.00034

**Published:** 2013-05-14

**Authors:** Jerome C. Wakefield

**Affiliations:** ^1^Department of Psychiatry and School of Social Work, New York UniversityNew York, NY, USA

Levy (2013) argues that “addiction is not a brain disease,” an important claim because, contrary to common wisdom, believing that mental disorders are brain diseases apparently increases stigma (Angermeyer and Matschinger, 2005; Schomerus et al., 2012). Levy presupposes the harmful dysfunction (HD) analysis of disorder (Wakefield, 1992a,b, 1999a,b, 2006): “[A]n individual suffers from a disorder only if they experience a biological dysfunction and that dysfunction is harmful, where the judgment of harm is made by reference to social norms of flourishing” (Levy, p. 11). He accepts that addicted individuals have substance-induced brain dysfunctions, and that when their dysfunctions cause harm (e.g., suffering, impairment of agency), such individuals are addictively disordered. (Note that throughout this commentary, consistent with HD and standard psychiatric usage, I use “disorder” as a generic term for medical pathology, inclusive of Levy’s term “disease.”)

Given these preliminary points, why does Levy then claim that addictive disorders are not brain diseases? Levy interprets the HD analysis as requiring that, to be a disorder, a dysfunction must not only cause harm but cause harm “in almost any accessible environment” (AAE) (p. 8); “[D]ysfunction plus impairment is not sufficient for disorder, when the impairment is due to social conditions that can relatively easily be altered” (p. 8). Levy observes that addicts sometimes abstain successfully or obtain safe, reliable drug access, suffering no harm. Thus, addictive disorder is not identifiable with brain dysfunction.

Why the AAE? Levy says it “is necessary to rule out conditions in which the appropriate response to suffering is to alter the environment and not to ‘treat’ the person” (p. 8). However, whether a condition is a disorder or not and whether treatment of the condition should be aimed at the person or the environment are two different questions. Many disorders are appropriately treated environmentally (e.g., dietary restriction in phenylketonuria, lowering episode-triggering expressed emotion in mentally ill individuals’ families).

Levy struggles with the many common disorder attributions that are apparent AAE counterexamples. A New Yorker’s pollen allergy and Arizona resident’s snake phobia are considered disorders, even if switching residences would alleviate both harms. Levy claims accessibility costs make such counterexamples “only apparent”; peanut allergies are disorders because “avoiding peanuts is, right now, far from costless” (p. 8). This defense of the AAE raises difficult questions about how costs are to be evaluated in deciding whether an environment is “easily altered” and an alternative environment “accessible.” It also potentially renders the AAE operationally meaningless because virtually any social change entails peanut-allergy-level costs.

To defend the AAE, Levy cites dyslexia, a presumed brain dysfunction impairing reading ability: “[I]f it is true that dyslexia was not a disease in the pre-literate past, because it did not cause an impairment…, then it seems that if it were possible costlessly to alter the environment so that it did not cause an impairment in sufferers today, it would not count as a disease today” (p. 9). Levy is not arguing that dyslexia is not a disorder today; rather, he is arguing that, as the AAE predicts, if there existed a costless way to alter the environment and eliminate dyslexia’s harm today, then, as in pre-literate times, dyslexia would not be a disorder today, either. This argument’s appeal as a defense of the AAE turns on an equivocation between actual versus counterfactual harmlessness. Pre-literate dyslexia was actually harmless, thus non-disordered; and if costless environmental changes were implemented that rendered dyslexia actually harmless today, then dyslexia would again be non-disordered. However, the AAE asserts the stronger claim that, if costless alterations to render dyslexia harmless did exist today, then even if they were not implemented and dyslexia remained quite harmful in our reading-demanding society, dyslexia would still not be a disorder simply because the possibility of such costless alterations means that an “easily accessible (possible) environment” would exist in which dyslexia would not be harmful. Nothing about pre-literate dyslexia’s status implies this counterintuitive conclusion that just the possibility of costlessly eliminating a dysfunction’s harm means that the dysfunction while it continues to cause harm is not a disorder. Our intuitive “disorder” concept that tracks actual harmful biological dysfunctions requiring our attention seems essentially abandoned by the AAE.

Nonetheless, the AAE suggests an important truth about the “harm” component of “disorder”: the social judgment that a condition is harmful may be based on misguided social values, and deeper judgments about what serves justice in the long run can override superficial harm judgments and thus negate disorder attributions. To this extent, my (1992) claim that harm is judged by social values was overly simplistic. For example, imagine that runaway slaves and Soviet dissidents (both claimed by respective social authorities to be disordered) had minor brain dysfunctions that made them less tolerant of oppression and more freedom-aspiring than others. These groups’ actions were socially judged as harmful by their societies, potentially justifying a disorder diagnosis if dysfunctions did exist. However, the attributions of harm were misjudgments (in our view and in the views of enlightened contemporaries) because the slaves’ and dissidents’ supposedly socially harmful actions were in fact warranted steps toward justice. Thus, even if they had such dysfunctions, no relevant harm and thus no disorder existed. The HD “harm” component, being normative, reflects deliberation about broader normative commitments, not just immediate social reactions. This seems close to Levy’s point: “[A]ddiction may not count as a disease because the suffering it causes is very largely due to social conditions that are, in some sense, optional” (p. 11). However, Levy stops short of attributing all addictive harm to social injustice.

Levy attempts to illustrate the usefulness of the AAE with an imagined example in which homosexuality turns out to be caused by a dysfunction, but still, he suggests, the AAE saves homosexuality from being a disorder because the harm is due to changeable socially oppressive attitudes. The example is problematic because, although horrifically oppressed, homosexuality’s purported harms justifying disorder attribution included features unrelated to oppression, such as the impossibility of having mutual biological children with the person one loves. The argument also falters if oppressive attitudes are not easily altered, as Levy admits. The process by which homosexuality actually did become depathologized illustrates not an appeal to the AAE but rather the sort of theoretical interaction of HD-harm with broader moral theory described above. Psychiatrists avoided the incendiary issue of whether homosexuality is caused by a dysfunction and instead overrode the traditional reproductive-harm value claim, arguing that what really matters from a values perspective is capacity for loving human relationships. Homosexual and heterosexual individuals are on all fours regarding this normative criterion for psychosexual health. Unlike the AAE, the value-theory-based approach allows depathologization of homosexuality even in circumstances of difficult-to-change attitudes or other costs.

Without the AAE, addictive disorders might be brain diseases even if brain dysfunctions only sometimes cause harm. Compare “addiction is a brain disease” with “tuberculosis is an infectious disease.” The latter is true, yet few people infected with tuberculosis develop disease because most people’s immune responses contain the infection. So, why is tuberculosis an infectious disease rather than, say, a disease of immune response in which the immune system does not successfully fight off the infection? The answer is that there is no known immune dysfunction in people who succumb to tuberculosis. The outcome seems due to an interaction of the infection with normal variations in immune system functioning. The individuation of the disorder is determined by the dysfunction (in this case the infection) that plays the largest role in explaining the symptoms, even when the disease occurs in only a minority of those with the dysfunction. Analogously, causal pathways to addictive disorders may involve an interaction between explanatory brain dysfunctions plus individual and environmental potentiating factors that are normal variations, thus making addiction a brain disorder.

However, a dysfunction that initiates the pathway to symptoms can be a risk factor for disorder rather than a disorder itself, if another dysfunction mediates between the initiating dysfunction and the ultimate symptoms, and if the mediating dysfunction better explains the symptoms. For example, BRCA-gene mutations increase breast cancer risk, but breast cancer is not a BRCA-gene disorder because further mutations must occur that directly explain breast cancer symptoms. Speculatively, this feature of the concept of disorder might suggest a different route by which to argue for Levy’s conclusion that addictions are not brain disorders. Instead of construing impairment of agency as one of addiction’s harms (as Levy does), one might argue that addictive disorders are dysfunctions of agency (Wakefield, 2009). If such dysfunctions of agency mediate between brain dysfunctions and symptoms, and if dysfunctions of agency best explain addictive symptoms, then one might argue that the addict’s brain dysfunction is indeed only a risk factor for disorder, not the addictive disorder itself.
